# Characterization and identification of cell death dynamics by quantitative phase imaging

**DOI:** 10.1117/1.JBO.27.4.046502

**Published:** 2022-04-28

**Authors:** Huai-Ching Hsieh, Po-Ting Lin, Kung-Bin Sung

**Affiliations:** a National Taiwan University, Department of Life Science, Taipei, Taiwan; b National Taiwan University, Department of Electrical Engineering, Taipei, Taiwan; c National Taiwan University, Graduate Institute of Biomedical Electronics and Bioinformatics, Taipei, Taiwan; d National Taiwan University, Molecular Imaging Center, Taipei, Taiwan

**Keywords:** quantitative phase imaging, cell death, morphology, time-series analysis

## Abstract

**Significance:**

Investigating cell death dynamics at the single-cell level plays an essential role in biological research. Quantitative phase imaging (QPI), a label-free method without adverse effects of exogenous labels, has been widely used to image many types of cells under various conditions. However, the dynamics of QPI features during cell death have not been thoroughly characterized.

**Aim:**

We aim to develop a label-free technique to quantitatively characterize single-cell dynamics of cellular morphology and intracellular mass distribution of cells undergoing apoptosis and necrosis.

**Approach:**

QPI was used to capture time-lapse phase images of apoptotic, necrotic, and normal cells. The dynamics of morphological and QPI features during cell death were fitted by a sigmoid function to quantify both the extent and rate of changes.

**Results:**

The two types of cell death mainly differed from normal cells in the lower phase of the central region and differed from each other in the sharp nuclear boundary shown in apoptotic cells.

**Conclusions:**

The proposed method characterizes the dynamics of cellular morphology and intracellular mass distributions, which could be applied to studying cells undergoing state transition such as drug response.

## 1 Introduction

Cell death identification and characterization are widely used in biological research. For example, in anticancer drug discovery, end-point observations such as colorimetric assays and protein analysis are performed to assess the cell death mechanisms.^1^ Specification of drug responses helps identify new drugs with unique mechanisms.^2^^,^^3^ In addition to end-point analysis, the progression of cell death, for example, the generation of extracellular vesicles during apoptosis, has also been investigated for a more comprehensive understanding of cell responses.^4^ Furthermore, real-time approaches for measuring changes in cellular status such as cytotoxicity^5^ and ATP levels^6^ at the single-cell level have been presented since cell heterogeneity heavily challenges some drug development processes.^7^ The prevalent real-time detection method is a fluorescence-based assay, which is specific and sensitive. However, most of them could only detect one to three targets and likely disturb end-point analysis such as flow cytometry and colorimetric assays due to the irreversible effects of fluorescent labeling and the interference between exogenous compounds.^8^ By contrast, label-free techniques are promising for overcoming these disadvantages and giving a proper characterization at the cellular level.^9^

Cellular changes during apoptosis have been measured by label-free methods *in vitro*, such as the localization of changes in NAD(P)H and FAD autofluorescence^10^ and the changes of cytochrome c distribution measured by Raman microscopy.^11^ Various label-free characteristics in two types of cell death were also investigated; Wang et al.^12^ demonstrated the increased NADH fluorescence lifetime in apoptosis but not in necrosis, and van der Meer et al.^13^ showed that the optical attenuation coefficient increased in apoptosis but decreased in necrosis using optical coherence tomography. Zhao et al.^14^ combined the multiphoton microscopy, optical coherence microscopy, and fluorescence lifetime imaging microscopy to classify apoptotic, necrotic, and normal cells in engineered tissues at 18 h post treatment. Although this study successfully identified two types of cell death, complicated and high-cost imaging systems are needed.

Quantitative phase imaging (QPI) is a label-free imaging techniques that records the phase distribution of a cell resulting from both the intracellular refractive index and cell thickness. The microscopic spatial resolution of QPI could provide information on both cellular morphology and intracellular mass dynamics, which could not be provided by the fluorescence-based method. Kühn et al.^15^ developed a label-free cytotoxicity screening assay to measure the cell viability by QPI and demonstrated that QPI is a potential tool for distinguishing normal cells from dead cells at the population level. Vicar et al.^16^ discriminated between apoptosis and necrosis based on QPI by training a long short-term memory (LSTM) network with manual labeling to predict the time-point of cell death and subsequently using the average values of two features within 10 h before the predicted death time as the inputs to a support vector machine (SVM) for classification. This research successfully distinguished the two kinds of cell death. However, the extraprocess of training LSTM is complicated and possibly introduces errors. Although averaging feature values over time reduces noise, the selection of the window width needs guidance and greatly influences the results. Moreover, this method provides only end-point features and not the dynamic change of features.

Our study applies QPI to simultaneously classify apoptotic, necrotic, and normal cells and characterize the dynamics of morphological and quantitative-phase features in the cell death process. Apoptosis and necrosis are two main types of cell death and are usually characterized according to different morphological changes: apoptosis is a caspase 3, 7-dependent programmed process with cell shrinkage whereas necrosis is an accidental cell death with cell swelling. We observe that most of the QPI features followed the sigmoid function during cell death and showed distinct alterations between apoptosis and necrosis. To quantify the dynamic changes of the features, we propose performing curve-fitting to the time-lapse features using the sigmoid function that adequately captures the dynamics of the features during cell death and reduces the effect of noise. The parameters of the sigmoid function represent the shape of the curve and have different biological meanings.^17^ Summarizing the dynamic changes of the features during cell death by these parameters is efficient and useful, for it is impracticable to show all time-lapse data directly. The parameters that show distinctions between apoptosis and necrosis are used to train an SVM classifier to identify the type of cell death. We report characteristics of the two types of cell death and the performance of the SVM classifier to demonstrate the usefulness of our proposed sigmoidal fitting method for analyzing QPI dynamics during cell death.

## 2 Methods

### 2.1 Diffraction Phase Microscopy

**Fig. 1:**
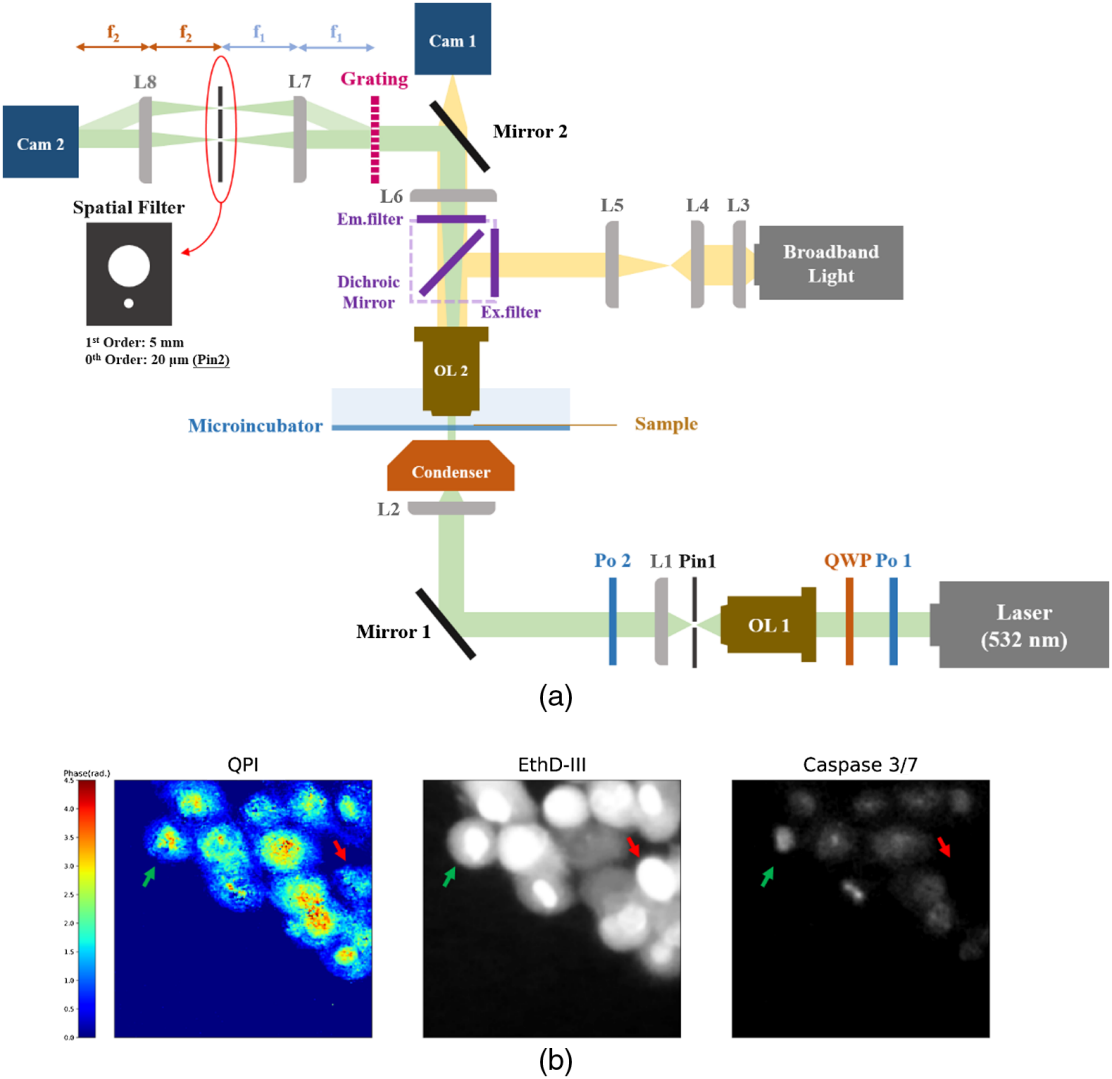
(a) Schematic diagram of the optical setup. Cam 1 is used to capture fluorescence images and Cam 2 is for QPI. The filter cube shown in violet can be moved out of the optical path for QPI. Po, polarizer; QWP, quarter waveplate; OL, objective lens; Pin, pinhole; L, lens; Cam, camera; f, focal length. (b) A quantitative phase image and corresponding fluorescent images (image size: 96  μm×96  μm; green arrow: an apoptotic cell; red arrow: a necrotic cell).

We used diffraction phase microscopy (DPM), a common-path QPI method that achieves highly stable phase imaging,^18^ to acquire quantitative phase images. A schematic diagram of our optical setup is shown in Fig. 1(a). A quasiplanar wavefront, generated by a 532-nm continuous-wave laser and an oil-immersion condenser, illuminates the sample at normal incidence with a $0.061\text{-}{\mathrm{cm}}^{2}$ beam size. The transmitted beam is collected by an objective lens, OL2 (Olympus UMPlanFI 20XW, 0.5 NA). A uniform reference beam is generated by a transmission grating (Edmund, 110  grooves/mm) and a pinhole (Edmund, 20  μm), and it interferes with the sample image on the sensor of a CMOS camera (GZL-CL-41C6M-C, Gazelle, Point Grey). Finally, quantitative phase images are obtained by off-axis holographic reconstruction and unwrapping methods.^19^^,^^20^ The transverse magnification of the DPM setup is about 46.3×, the theoretical lateral resolution of the imaging system with coherent illumination is 0.82λ/NA=0.87  μm,^21^ the pixel-size in quantitative phase images is 0.48  μm, and fluorescence microscopy is combined with the DPM to distinguish different types of cells. The optical power density at the sample plane is $131\;\mu W/{\mathrm{cm}}^{2}$, which is lower than the power density used in a previous study that reported time-lapse live cell imaging without phototoxicity.^22^

### 2.2 Cell Culture, Hydrogen Peroxide Treatment, and Time-Lapse Imaging

Human retinal pigment epithelium cell line, hTERT-RPE-1 (American Type Culture Collection, ATCC), was chosen as the model cell. hTERT-RPE-1 cells were grown into a monolayer in Dulbecco’s modified eagle medium: nutrient mixture F-12 (Life Technologies) supplemented with 10% fetal bovine serum (Cytiva) and 1% antibiotic-antimycotic (GeneDireX) at 37°C, and 5% ${\mathrm{CO}}_{2}$.

The treatment of 600 to 700  μM $H_{2}O_{2}$, a common inducer of cell death,^23^ was used to induce apoptosis and necrosis in hTERT-RPE-1 cells. After treating the cells for 1.5 h, we stained the cells by CellEvent Caspase-3/7 Green Detection Reagent (Caspase-3/7, ThermoFisher Scientific) and Ethidium Homodimer III (EthD-III, Biotium), a DNA dye for dead cells only. Apoptotic cells show both dyes positive, necrotic cells show Caspase-3/7 negative and EthD-III positive, and normal cells show both dyes negative under fluorescence microscopy. Time-lapse quantitative phase images were acquired every 6 min for 2 to 4 h after 30 min of staining, and fluorescent images were captured at the beginning and end of time-lapse imaging to provide the ground-truth type of cell death [Fig. 1(b)]. The total exposure time was less than three hours during the time-lapse experiment. A separate viability test was performed on cells illuminated by a $131\;\mu W/{\mathrm{cm}}^{2}$ of 532 nm laser for 3 h. On average, 94.4% of the cells were viable (see Supplementary Material for details). Therefore, phototoxicity effects could be excluded.

### 2.3 Cell Segmentation, Cell Tracking, and Feature Extraction

Individual cells in the quantitative phase images were segmented by U-Net,^24^ the watershed algorithm,^25^ and manual corrections sequentially. U-Net was used for foreground–background separation, the watershed algorithm was applied to distinguish each cell, and manual corrections were applied to cells that were too densely located to be separated automatically. Cell tracking was then performed by finding the masks with the largest overlapping area in two consecutive frames.^26^

**Fig. 2:**
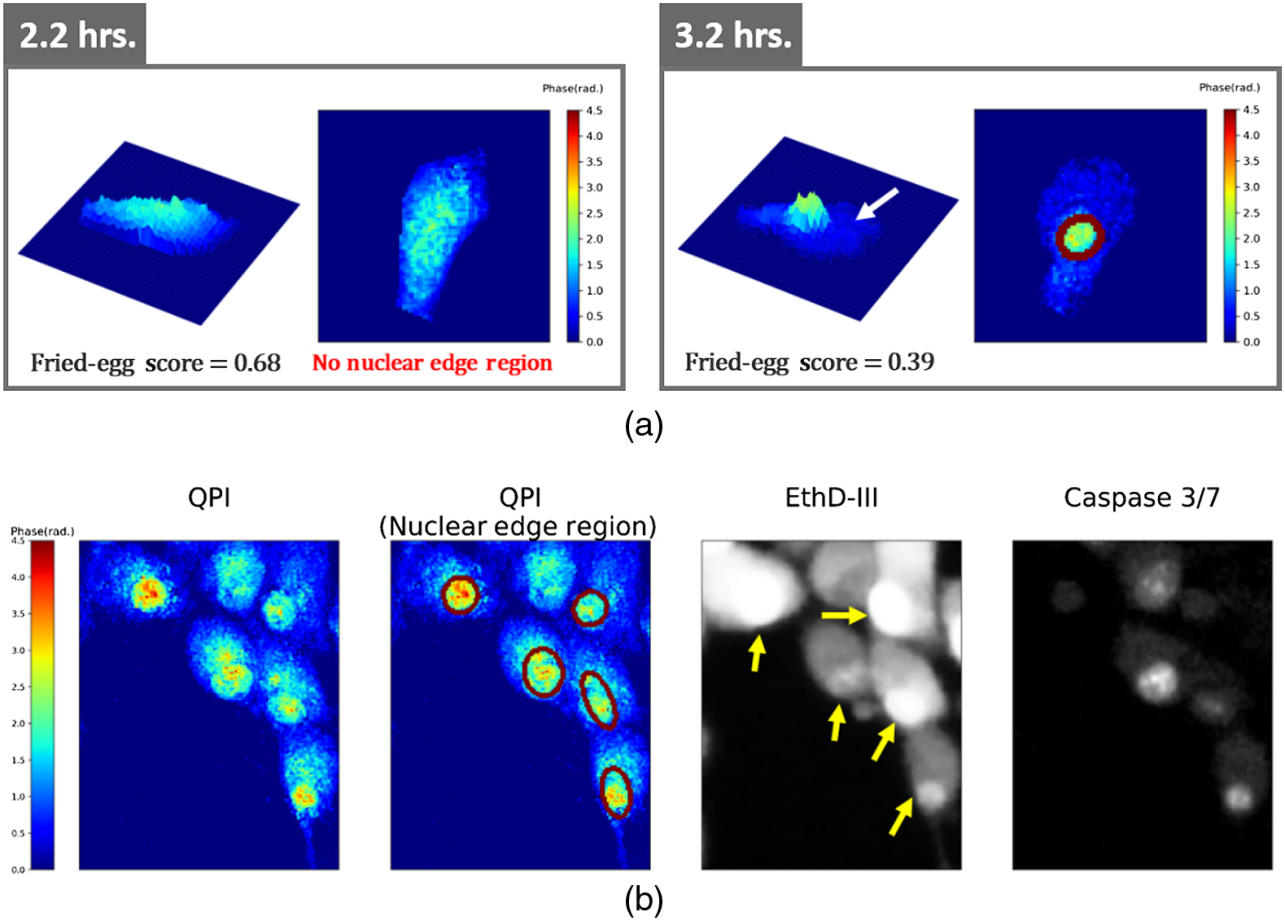
(a) The apoptotic volume decrease in the peripheral region (white arrow) and the sharp boundary of the nucleus (red ellipse) in an apoptotic cell (the cell is cropped. Image size: 38.4  μm×38.4  μm). (b) Nuclear edge regions (red ellipses) identified in QPI of apoptotic cells are roughly consistent with nuclei (yellow arrows) in the EthD-III-stained fluorescent image. (Image size: 91.2  μm×72  μm).

Eleven features were extracted from individual cells as summarized in Table 1. Four features describe cell morphology: cell area, circularity, eccentricity, and solidity. Circularity indicates the degree of roundness, and the eccentricity is defined as the ratio of the minor axis to the major axis lengths of an ellipse fitted to a cell. Solidity, describing the cellular protrusions, is the ratio of the cell area to the smallest convex hull area covering the cell. The other seven features are quantitative-phase features. Optical volume represents the integrated phase over the cell area, which is proportional to the dry mass of the cell.^27^ To analyze the intracellular distribution of the phase, Otsu thresholding and morphological closing were used to separate a central connected region in a cell from its peripheral region. The central region was defined as a connected region with its phase higher than 1.1 times of the Otsu threshold. The mean phase in the central region, the mean phase in the peripheral region, and the ratio between them were also analyzed. The fried-egg score, defined as the proportion of the central region area to the cell area, was used to assess the apoptotic volume decrease^28^ in the peripheral region of a cell as shown in Fig. 2(a). The nuclear edge score was used to quantify the average amplitude of the phase gradient around the sharp boundary of the nucleus observed in apoptotic cells. It was defined as the average phase gradient over a two-pixel wide nuclear edge region identified by ellipse fitting and edge detection. Examples of automatically identified nuclear edge regions in apoptotic cells and corresponding fluorescent images are shown in Fig. 2(b).

**Table 1:** Definition of features extracted from quantitative phase images.

**Morphological features**			
Cell area	Circularity	Eccentricity	Solidity
Cell mask area	$4\pi \frac{\text{cell area}}{{\text{cell perimeter}}^{2}}$	$\frac{\text{Minor axis length}}{\text{Major axis length}}$	$\frac{\text{Cell area}}{\text{Cell convex hull area}}$
**Whole-cell phase features**			
Δϕ(x,y): phase at the pixel(x,y),			
Δx,Δy: pixel size in the QPI			
Optical volume		Standard deviation of cell phase	
$\underset{(x,y)\in \text{Cell}}{\sum }\Delta \phi (x,y)\frac{\lambda }{2\pi }\Delta x\Delta y$		$\sqrt{\frac{\underset{(x,y)\in \text{Cell}}{\sum }{\left|\Delta \phi (x,y)-\overset{¯}{\Delta \phi (x,y)}\right|}^{2}}{\text{number of pixels}}}$	
**Intracellular phase distribution features**			
C: the main closed region in the cell with phase>1.1*threshold			
P: cell region−C			
Mean of central phase	Mean of peripheral phase	$\frac{\text{Peripheral phase}}{\text{Central phase}}$	Fried-egg score
$\frac{\underset{(x,y)\in C}{\sum }\Delta \phi (x,y)\Delta x\Delta y}{\text{Area of }C}$	$\frac{\underset{(x,y)\in P}{\sum }\Delta (x,y)\Delta x\Delta y}{\text{Area of }P}$	$\frac{\text{Mean peripheral phase}}{\text{Mean of central phase}}$	$\frac{\text{Area of }C}{\text{Cell area}}$
Nuclear edge score		1. Define a high gradient region H within C by Ostu thresholding of the gradient magnitude in C.	
		2. Find the two largest ellipses within H and rank them by the average gradient magnitude over a two-pixel wide edge region of the ellipse.	
$\left\{ \begin{array}{ll} \frac{\underset{(x,y)\in N}{\sum }|\nabla (\Delta \phi (x,y))|\Delta x\Delta y}{\text{Area of }N}, & \text{if }N\text{ exists}\\ 0, & \text{else} \end{array}\right.$		3. Check the two candidate ellipses if $0.1<\frac{\text{ellipse area}}{\text{cell area}}<0.3$, $\frac{\text{major axis}}{\text{minor axis}}$ <2.5, and the average gradient magnitude >0.6× the mean of cellular phase.	
N: the nuclear edge region		4. N is the nuclear edge region of the highest-ranked ellipse that passes the condition check in step 3.	

### 2.4 Sigmoidal Fitting and Parameter Extraction

We performed the sigmoidal fitting within a window length of 13 time-points by linear least-squares optimization. Feature values were normalized before the sigmoidal fitting in each window as follows:

$$\left\{ \begin{array}{ll} x_{\text{normalized}}=\frac{x-x_{1}}{x_{1}}, & \text{for }{\mathrm{features}}^{*}\\ x_{\text{normalized}}=x-x_{1}, & \text{else} \end{array},\right.$$ (1)

where $x_{1}$ is the first feature value in the window, x is the original data, $x_{\text{normalized}}$ is the normalized data, and ${\text{features}}^{*}$ include the optical volume, cell area, mean of central phase, and mean of peripheral phase.

The sigmoid function centered around the time-point *Cutoff* is expressed as

$$f(t)=\frac{\text{Amplitude}}{1+e^{\text{Gain}(\text{Cutoff}-t)}},$$ (2)

where *Amplitude* represents the difference between the final value and the initial value, and *Gain* indicates the rate of the change. A positive *Amplitude* represents an increase of the feature, and a negative *Amplitude* indicates a decrease of the feature.

To find a proper range of time-points for performing the sigmoidal fitting, we determined the most likely time-point of feature change for each cell by selecting the *Cutoff* when most features showed a reasonably good fit to the sigmoid function:

$$T=k{|}_{\max({\text{count}}_{k})},\;{\text{count}}_{k}=\overset{11}{\underset{i=1}{\sum }}{\text{fit}}_{ik},\;{\text{fit}}_{ik}=\{\begin{array}{ll} 1, & \text{if }R_{ik}^{2}\geq 0.7\\ 0, & \text{else} \end{array},$$ (3)

where T is the selected time-point in hours, ${\text{count}}_{k}$ is the number of features showing a good sigmoidal fit for the Cutoff=k, and $R_{ik}^{2}$ represents the goodness of the least-squares fit for the i’th feature with Cutoff=k. The threshold of 0.7 for $R_{ik}^{2}$ was used to exclude poor fit due to noise or fluctuations in cellular morphology and mass distribution. Furthermore, since some of the features changed later than others during cell death, the final sigmoidal fit of each feature was determined by finding the *Cutoff* within the time range of T and T+2 that resulted in the maximum |Amplitude×Gain| for the feature.

## 3 Results

### 3.1 Characterization of Cell-Death Features

**Fig. 3:**
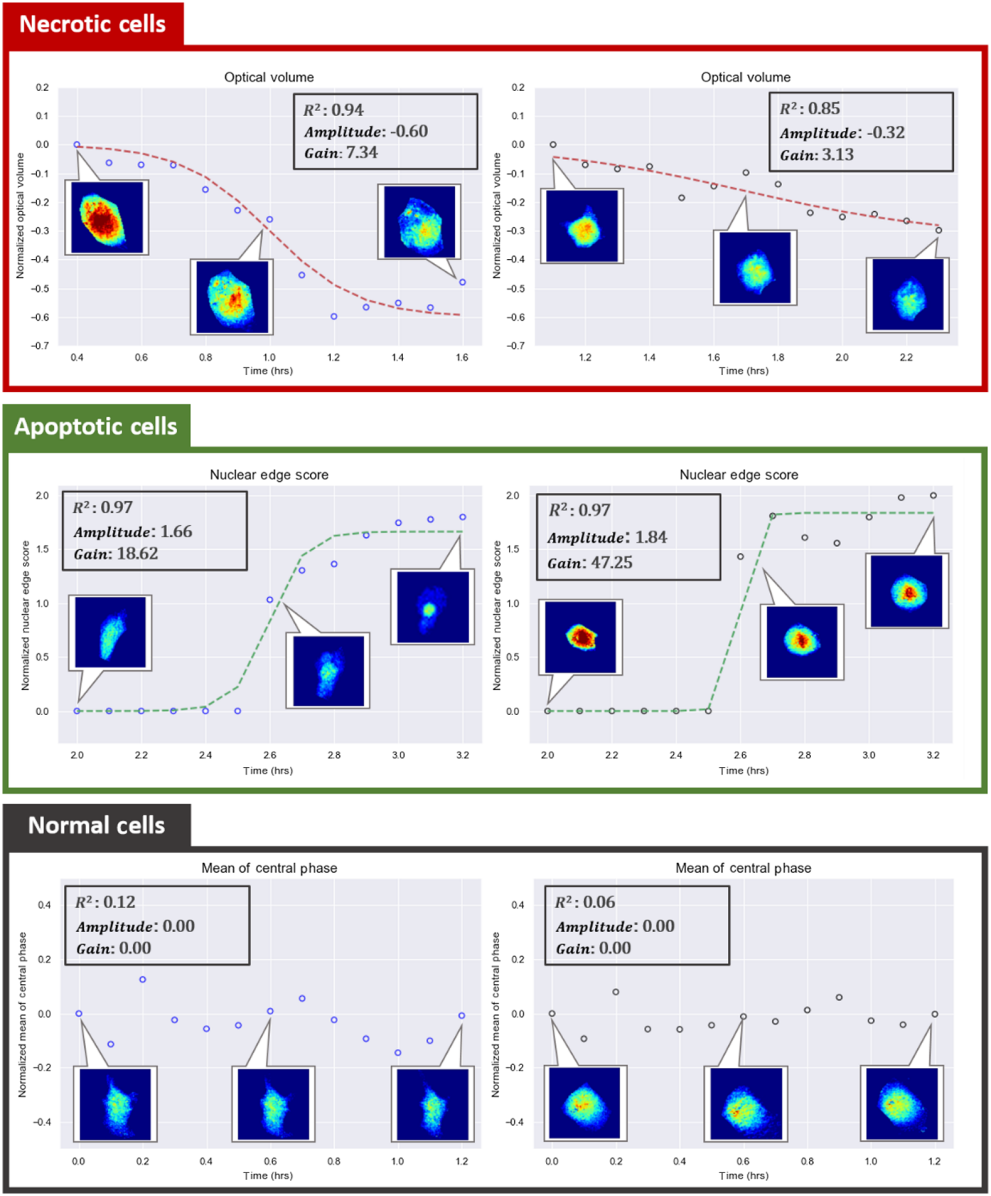
Examples of decreases of the optical volume in two necrotic cells, increases of the nuclear edge score in two apoptotic cells, and small fluctuations of the mean of the central phase in two normal cells with the corresponding *Amplitude*, *Gain*, and $R^{2}$. (All cells are cropped, image size: 38.4  μm×38.4  μm. Color bar: 0 to 4.5 rad).

**Fig. 4:**
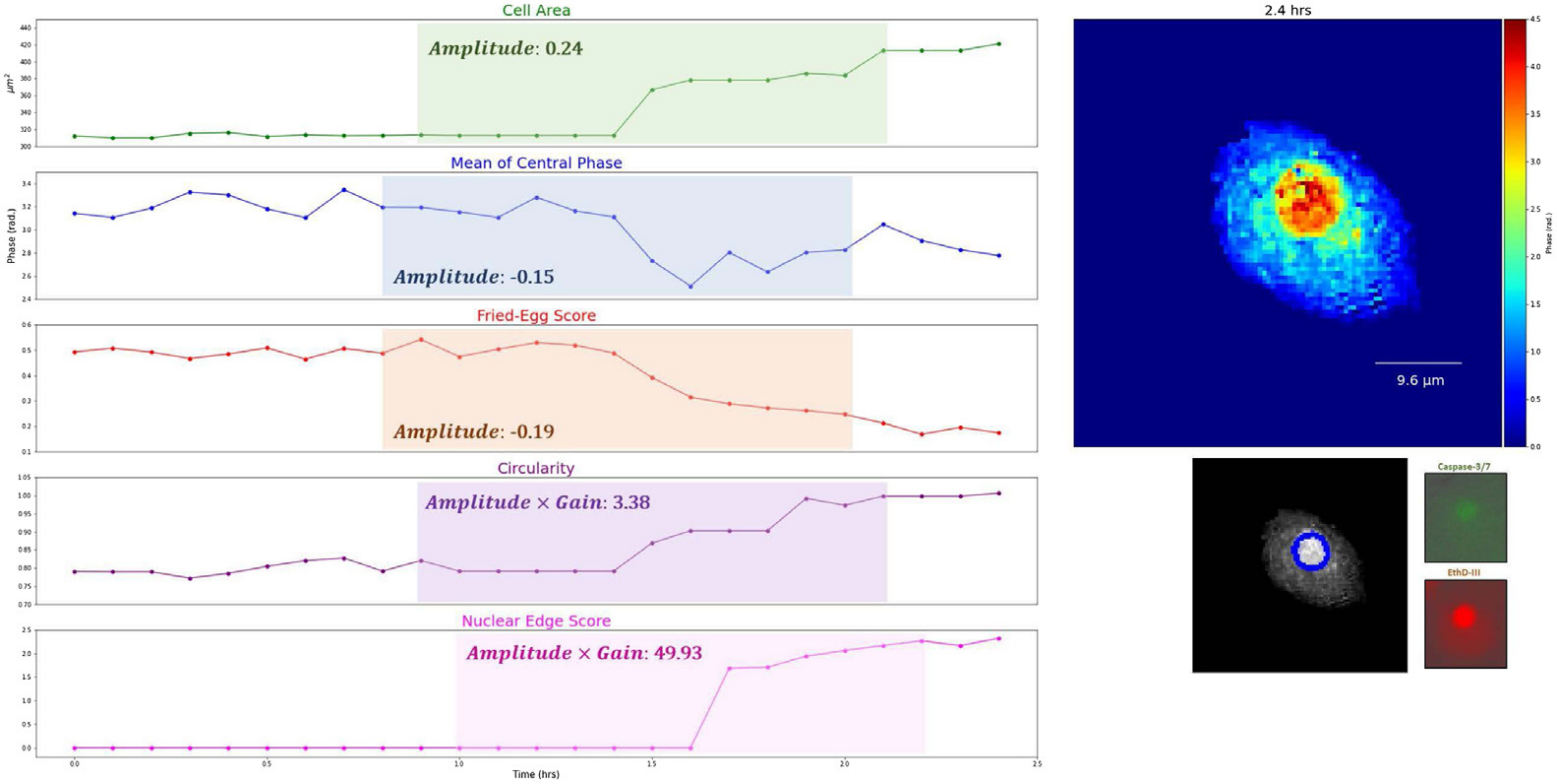
Another example of time-lapse phase images (blue ellipses represent nuclear edge regions) with the dynamics of selected features in typical apoptosis (SVM error rates <10%, see Sec. 3.2 for details; the cell is cropped) (Video 1, MP4, 4.0 MB [URL: https://doi.org/10.1117/1.JBO.27.4.046502.1]).

**Fig. 5:**
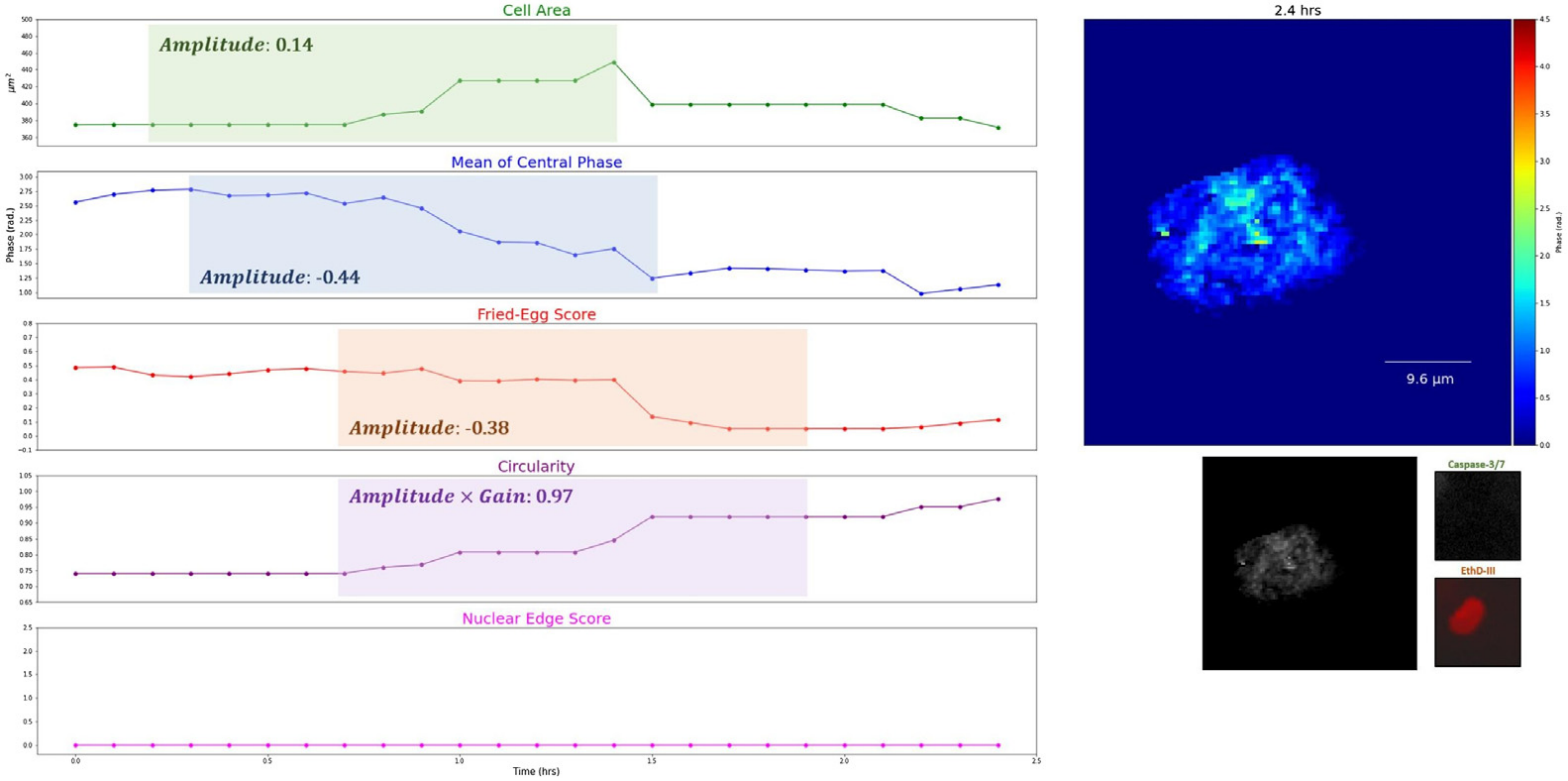
Another example of time-lapse phase images with the dynamics of selected features in typical necrosis (SVM error rates <10%, see Sec. 3.2 for details; the cell is cropped) (Video 2, MP4, 4.0 MB [URL: https://doi.org/10.1117/1.JBO.27.4.046502.2]).

**Fig. 6:**
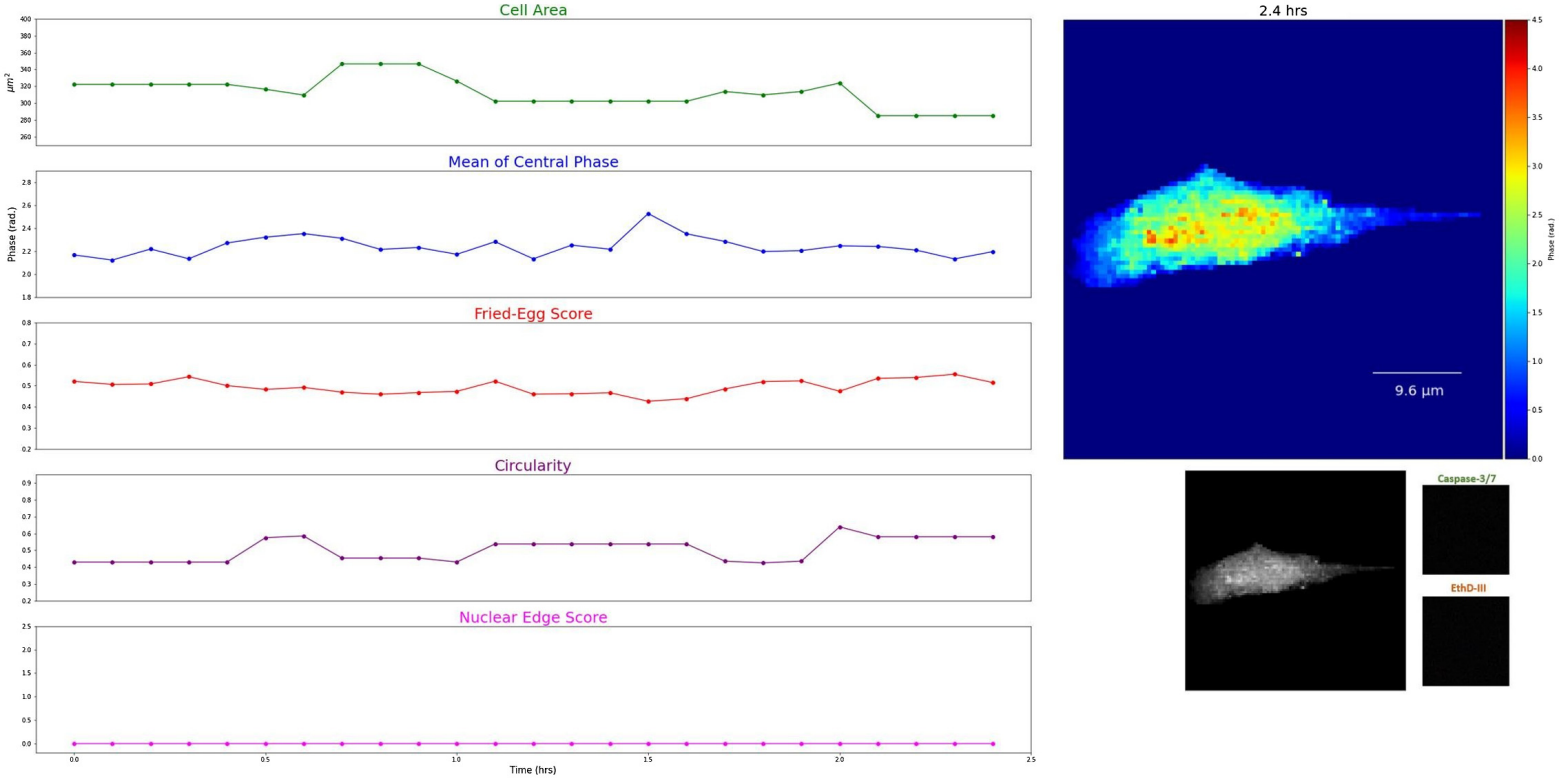
Another example of time-lapse phase images with the dynamics of selected features in a normal cell (the cell is cropped) (Video 3, MP4, 3.2 MB [URL: https://doi.org/10.1117/1.JBO.27.4.046502.3]).

The data consisted of 149 normal cells, 103 apoptotic cells, and 103 necrotic cells as determined by end-point fluorescence imaging. Examples of extracted features of time-lapse images and the corresponding *Amplitude*, *Gain*, and $R^{2}$ are shown in Figs. 3–6. The sigmoidal fitting results of apoptotic and necrotic cells clearly showed good matches to the transition of feature values. The average $R^{2}$ of the fitting results above the threshold of 0.7 was 0.85 over all features in apoptotic and necrotic cells, and the standard deviation was 0.079, indicating good fits of the time-lapse course of the features by the sigmoid function. On average, each apoptotic cell and necrotic cell showed obvious sigmoidal changes in 7.23 and 6.92 features, respectively, out of the 11 analyzed with a standard deviation of 1.71 and 1.76 features, respectively. By contrast, each normal cell showed sigmoidal changes in an average of only 0.95 features with a standard deviation of 1.28. Distributions of the number of features with sigmoidal changes per cell are plotted for the three types of cells in Fig. S1 in the Supplementary Material. The results confirm our observation that QPI features follow a sigmoid function during cell death.

**Fig. 7:**
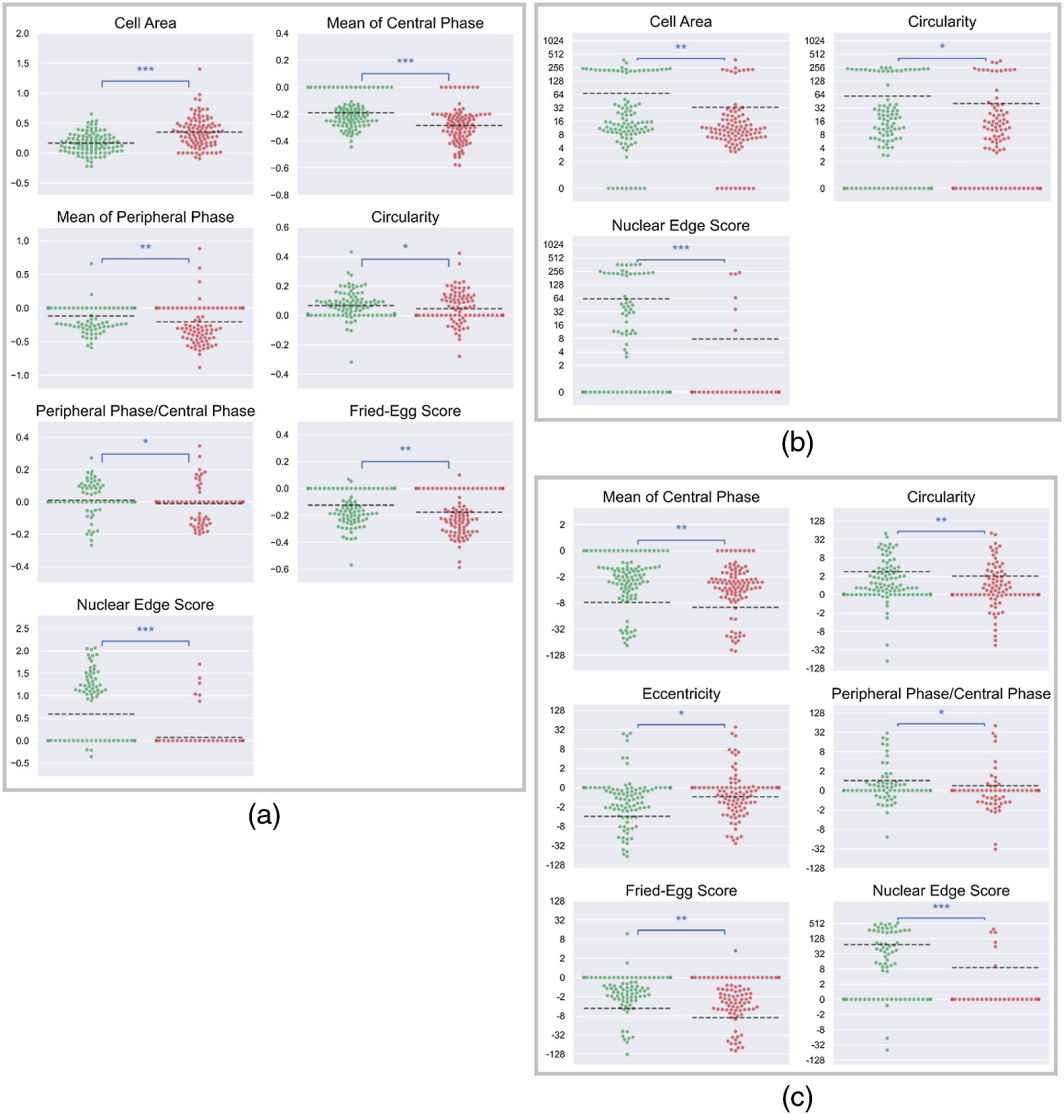
Distributions of (a) *Amplitude*, (b) *Gain*, and (c) Amplitude×Gain of features with statistical significances between apoptotic cells and necrotic cells (green dots: apoptotic cells; red dots: necrotic cells; *: statistically significant difference p value<0.05; **: statistically significant difference p value<0.01; ***: statistically significant difference p value<0.001; dashed line: the average value).

To investigate differences in the features that could help discriminate between apoptotic and necrotic cells, we performed the Wilcoxon rank-sum test on *Amplitude*, *Gain*, and Amplitude×Gain of the 11 features. Figure 7 shows swarm plots of the feature values showing significant differences (p<0.05) between the two types of cell death. On average, necrotic cells decreased more in the mean of central phase, mean of peripheral phase, and fried egg score, whereas apoptotic cells increased more in circularity and the nuclear edge score. Necrotic cells also showed larger increments but slower changes in cell area than apoptotic cells. In addition to the two shape-related parameters, *Amplitude* and *Gain*, the product of them represents the combination of both the extent and rate of changes and shows larger differences between the two types of cell death in eccentricity and nuclear edge score. Furthermore, the *Amplitude* of several features such as the mean of peripheral phase, fried-egg score, and nuclear edge showed bimodal distributions with one of the peaks near zero, indicating that some cells showed little or no change in these features. By contrast, decreases in the mean of central phase and increases in cell area were more commonly seen in the two types of cell death.

### 3.2 Identification and Classification of Cell Death

A nonlinear SVM model was trained to classify normal, apoptotic, and necrotic cells based on selected parameters of the sigmoidal fitting according to the Wilcoxon rank-sum test results. All of the parameters were processed by gamma correction and normalization before training. The data consisting of 355 cells with each cell’s classification by the end-point fluorescence imaging were split into training, validation, and test data sets in a ratio of 2:1:2 for each classification. The validation sets were used for parameter optimization, and the model performance was averaged over 500 random splits of data to reduce bias resulting from the choice of testing sets.^29^

**Fig. 8:**
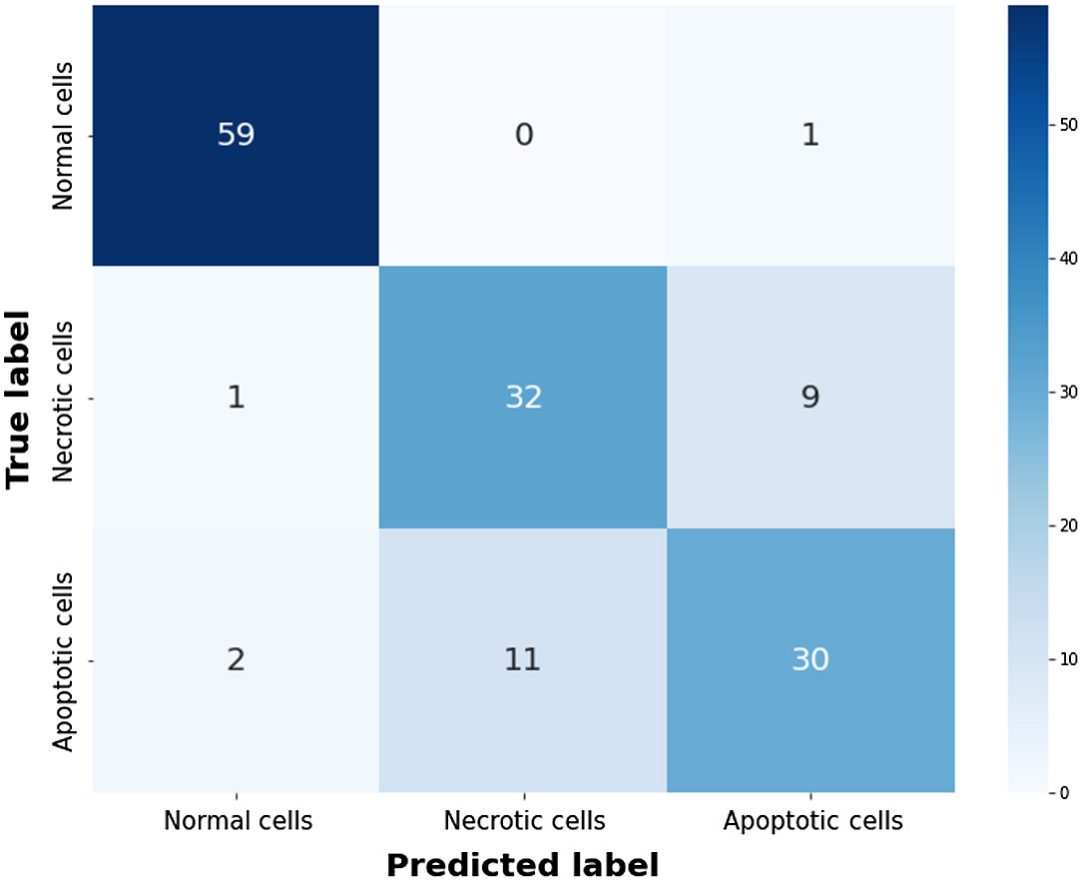
Confusion matrix of our SVM model to classify three types of cells with 84.0% overall accuracy on an average of 500 times.

The overall accuracy achieved for classifying normal, apoptotic, necrotic cells was 84.0%. The parameters selected to build the optimal classifier included *Amplitude* of the cell area, mean of central phase, and fried-egg score and Amplitude×Gain of circularity and nuclear edge score. The confusion matrix obtained by averaging the results of the 500 repeats is shown in Fig. 8. If the two types of cell death were combined, the accuracy of discriminating normal cells from dead cells was 97.2%. We also trained a second SVM classifier to distinguish between apoptosis and necrosis using *Amplitude* of the cell area, mean of peripheral/central phase, and fried-egg score and Amplitude×Gain of nuclear edge score. The accuracy was 77.3%, which was within the range of the results from several cell lines and treatments in a previous study using LSTM and SVM.^16^

**Fig. 9:**
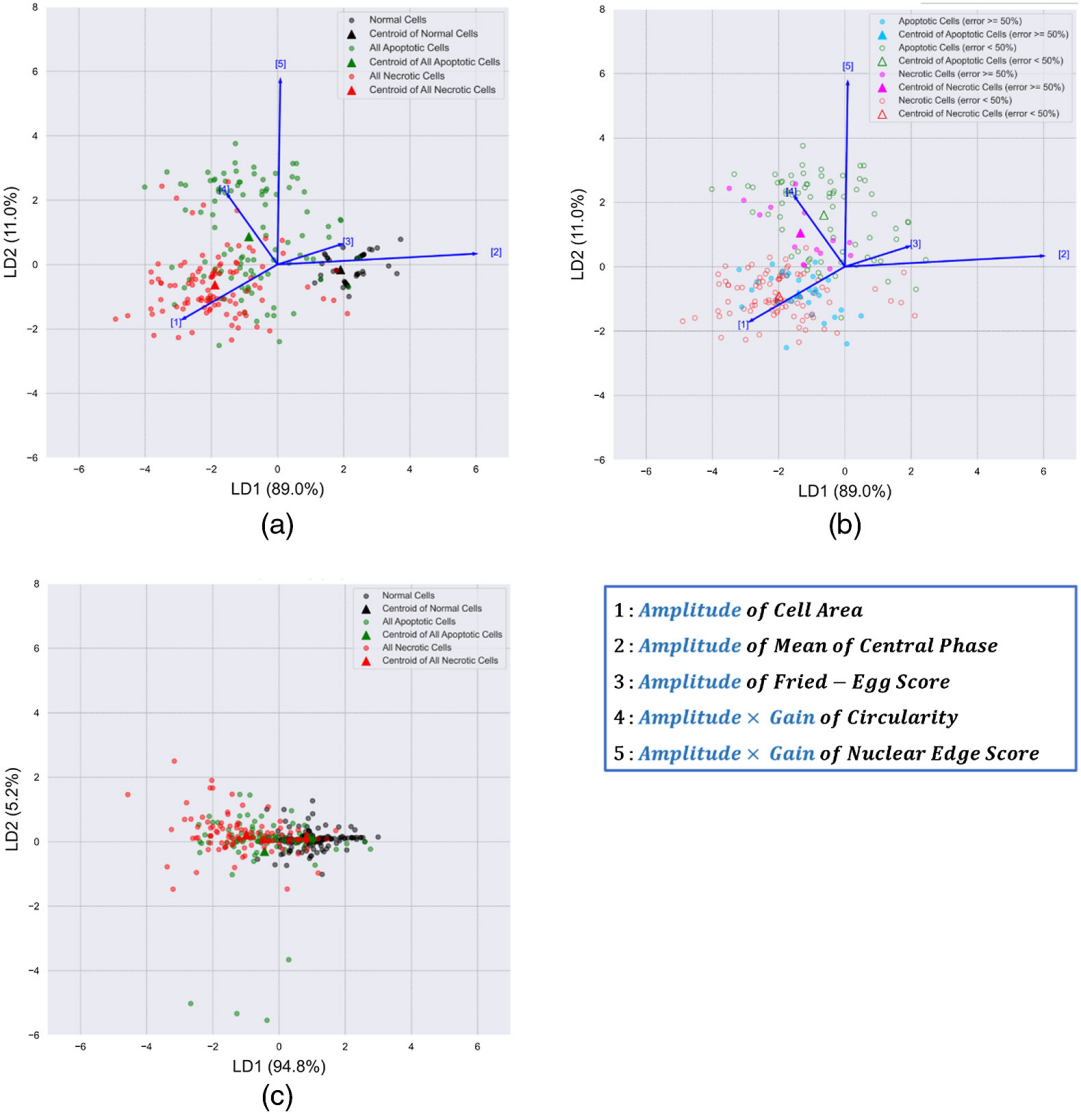
(a) The LDA results of *Amplitude* of the cell area, mean of central phase, and fried-egg score and Amplitude×Gain of circularity and nuclear edge score in normal (black), apoptotic (green), and necrotic (red) cells. (b) The cells with higher SVM error rates (>=50%) were annotated with different colors for apoptosis (cyan) and necrosis (magenta). (c) LDA results of manually selecting time-points to calculate changes in the cell area, mean of central phase, fried-egg score, circularity, and nuclear edge score.

To get more insights about morphological and QPI characteristics that separate the three classes of cells, we performed a linear discriminant analysis (LDA) of the parameters selected to train the optimal SVM classifier. As shown in Fig. 9(a), the main differences between the three groups are changes in the mean of central phase and the nuclear edge score, with changes in the cell area and circularity contributing secondarily. Although normal cells are well separated from the two types of cell death, there are substantial overlaps between necrotic and apoptotic cells. We then plot apoptotic and necrotic cells that were misclassified more than half of the time (error rate >50%) in different symbols from those classified correctly more than half of the time (error rate <50%). Figure 9(b) shows that the centroid of the apoptotic cells with high error rates (cyan symbols) is very close to the centroid of necrotic cells with low error rates (red symbols). In other words, the former shows lower Amplitude×Gain of the nuclear edge score that is characteristic of necrotic cells and, hence, cannot be classified correctly based on QPI features. Moreover, apoptotic cells spread over a wider area than the other two types of cells as shown in Fig. 9(a), suggesting a more diverse change of features in apoptosis. Our finding that only about half of the apoptotic cells show obvious nuclear edge is consistent with previous research.^16^

The efficacy of using sigmoidal curve fitting to characterize cell death can be further demonstrated by comparing our LDA results with simply taking the difference of feature values between manually selected time-points. Figure 9(c) shows LDA results of changes in the cell area, mean of central phase, fried-egg score, circularity, and nuclear edge score between manually chosen final and initial time-points. The LDA results using the sigmoidal fitting method show better separations between the three classes, suggesting superior characterization and classification. Although simple subtraction can extract changes in the feature values, selecting the time-points is subjective and susceptible to noise or random fluctuations of the features. Moreover, sigmoidal fitting quantifies the rate of change through the parameter *Gain*, which is not available from simple subtraction.

## 4 Discussion and Conclusion

Although QPI has been widely used to image many types of cells under various conditions, the dynamics of QPI features during cellular processes such as cell death have not been thoroughly quantified. We proposed exploiting curve fitting which is widely used to extract trends of dynamic cellular characteristics in response to environmental stimuli or during state transition such as in seed germination^30^ and gene expression analysis.^31^^,^^32^ The selection of the sigmoid function to fit the QPI dynamics is based on our observation and supported by previous research.^16^^,^^33^ The parameters extracted from fitting the time-lapse QPI features mainly reflect cell mass dynamics. Single-cell mass dynamics obtained by a cantilever-based microfluidic mass sensor have been used to assay the differential drug response and sensitivity due to different gene expressions.^9^ The proposed QPI and sigmoidal fitting method quantifies single-cell mass dynamics with the advantages of high throughput and providing additional intracellular mass dynamics.

We also demonstrated the use of new features to quantify intracellular mass dynamics during cell death. The phase of a pixel in a quantitative phase cell image is proportional to the dry mass density integrated over the thickness of the cell at that pixel. Since epithelial cells are typically thicker at and around the nucleus, the parameter “mean of central phase” represents the mean dry mass per unit area of the region containing the nucleus and nearby organelles, such as endoplasmic reticulum, Golgi apparatus, and mitochondria. Necrotic cells showed a larger decrease in the mean of the central phase than apoptotic cells, possibly due to the dysfunction and/or degradation of organelles in the central region; apoptotic cells showed a more frequent increase in the nuclear edge score, which quantified the sharper boundary of the nucleus. Moreover, the heterogeneity in each type of cell death was evidenced in the results of the single-cell analysis (Figs. 7 and 9). Therefore, we suggest that our method could not only be applied to the identification of apoptosis and necrosis but also be used to help specify responses of cells to treatments such as drug candidates and to elucidate possible subcellular kinetics.

The proposed sigmoidal fitting to QPI features could also help quantify and summarize single-cell dynamics in other applications such as investigating the cellular response to the treatment with drug-delivering nanographene, which alters the intracellular quantitative phase distribution after nanoparticle uptake.^34^ In addition, the investigation of drug responses in suspended cells also plays an essential role in some clinical uses such as evaluating chemotherapeutic sensitivity. Xin et al.^35^ reported a high-throughput screening method for assessing the drug resistance of epithelial ovarian cancer cells using the combination of QPI and microfluidic devices. The results showed that drug-resistant cancer cells differed in morphological and cellular phase features from drug-sensitive ones. We suggest the sigmoidal fitting method might also be used to capture dynamics of suspended cells in a relatively short period of time for high-throughput detection applications.

Compared with the study of Vicar et al.,^16^ our sigmoidal fitting and windowing method obtains the time-point of cell death along with dynamics of features, which eliminates an extraprocess of training a neural network to predict the cell death time-point. Moreover, in the study of Vicar et al.,^16^ the authors only showed example feature curves of a few cells and did not report systematic quantification of the feature dynamics in the apoptotic and necrotic cells populations. By contrast, we demonstrated a reliable curve fitting method to parameterize and quantify the dynamics of cell morphology and intracellular mass distribution from QPI of live cells during cell death. Importantly, Sigmoidal fitting captures the trend in feature changes and is less influenced by fluctuations in cellular morphology or intracellular mass distribution, as evidenced by results shown in Sec. 3.1 and Figs. 9(a) and 9(c). Our approach summarizes the complicated feature curves into simple parameters representing the extent and rate of changes, which enables further analysis and comparison of cell dynamics heterogeneity at the population level. As a demonstration, we report quantified changes of many features in $H_{2}O_{2}$-induced apoptosis and necrosis in hTERT-RPE-1 cells. Therefore, our method robustly provides quantitative metrics to summarize the dynamics of apoptosis and necrosis instead of classification only.

In conclusion, this study proposes a label-free method based on QPI to identify apoptosis and necrosis and characterize cell death dynamics using the sigmoidal fitting with parameters that quantify both the extent and rate of feature changes. SVM classifiers based on the sigmoidal parameters showed 84.0% overall accuracy in the classification of normal cells and the two types of cell death and 77.3% accuracy in the discrimination of only apoptosis and necrosis. This label-free, single-cell technique is promising for the characterization of the dynamics of cellular morphology and intracellular mass distribution on cells undergoing state transition, and it has the potential to aid in drug development.

## Code, Data, and Materials Availability

Request of code, data and materials presented in this paper should be directed to the corresponding author at kbsung@ntu.edu.tw.
