# Supplementary material for: Characterization and identification of cell death dynamics by quantitative phase imaging
Source: J Biomed Opt. 2022 Apr 28;27(4):046502. doi: 10.1117/1.JBO.27.4.046502 (PMC9047449; doi:10.1117/1.JBO.27.4.046502)
Supplement: Supplementary file 1 [file JBO_027_046502_SD001.pdf]

Cell Viability Test for Photo-toxicity

4,000 hTERT-RPE-1 cells were seeded in each 96-well plate, and after 17 hours, the experimental groups were illuminated at 131 μW/cm^2 for three hours in the micro incubator in our optical system. The control groups were cultured in the traditional cell incubator simultaneously. The cell viability test was performed using Cell Counting Kit-8 (CCK-8, MedChemexpress) after the light treatment, and the cell viability of each experimental group was normalized to the control group. Cells illuminated at 131 μW/cm^2 for three hours showed cell viability at 94.4% with a standard deviation of 2.3%.

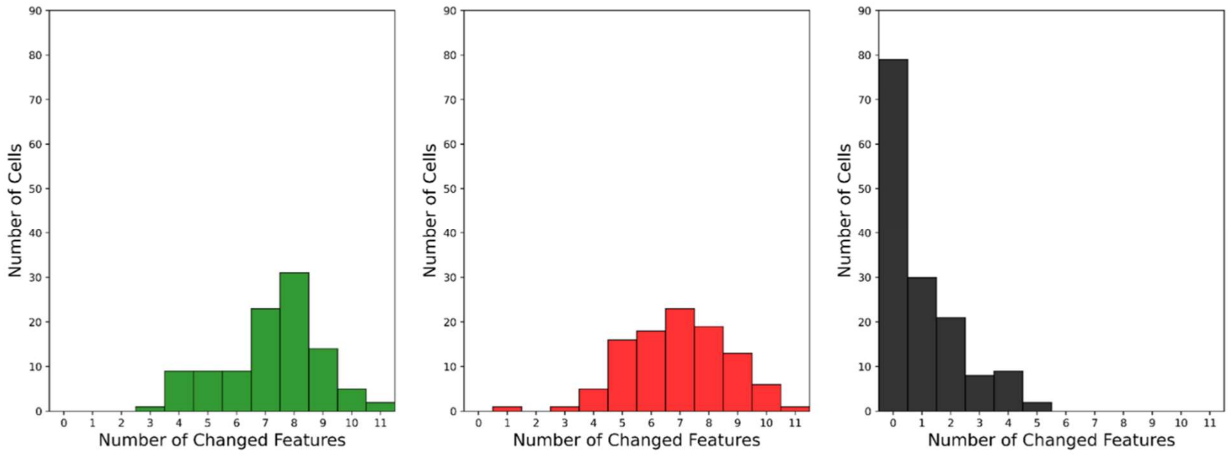

(a) (b) (c)

Fig. S1 Distributions of the number of features that showed sigmoidal changes in (a) apoptotic cells (b) necrotic cells (c) normal cells
